# Growth inhibition of *Spodoptera frugiperda* larvae by camptothecin correlates with alteration of the structures and gene expression profiles of the midgut

**DOI:** 10.1186/s12864-021-07726-8

**Published:** 2021-05-26

**Authors:** Benshui Shu, Yan Zou, Haikuo Yu, Wanying Zhang, Xiangli Li, Liang Cao, Jintian Lin

**Affiliations:** Guangzhou City Key Laboratory of Subtropical Fruit Trees Outbreak Control, Institute for Management of Invasive Alien Species, Zhongkai University of Agriculture and Engineering, 313 Yingdong teaching building, 510225 Guangzhou, PR China

**Keywords:** *Spodoptera frugiperda*, Camptothecin Adverse effects, Transcriptome analysis

## Abstract

**Background:**

*Spodoptera frugiperda* is a serious pest that causes devastating losses to many major crops, including corn, rice, sugarcane, and peanut. Camptothecin (CPT) is a bioactive secondary metabolite of the woody plant *Camptotheca acuminata*, which has shown high toxicity to various pests. However, the effect of CPT against *S. frugiperda* remains unknown.

**Results:**

In this study, bioassays have been conducted on the growth inhibition of CPT on *S. frugiperda* larvae. Histological and cytological changes were examined in the midgut of larvae fed on an artificial diet supplemented with 1.0 and 5.0 µg/g CPT. The potential molecular mechanism was explored by comparative transcriptomic analyses among midgut samples obtained from larvae under different treatments. A total of 915 and 3560 differentially expressed genes (DEGs) were identified from samples treated with 1.0 and 5.0 µg/g CPT, respectively. Among the identified genes were those encoding detoxification-related proteins and components of peritrophic membrane such as mucins and cuticle proteins. Kyoto Encyclopedia of Genes and Genomes (KEGG) pathway enrichment analyses indicated that part of DEGs were involved in DNA replication, digestion, immunity, endocrine system, and metabolism.

**Conclusions:**

Our results provide useful information on the molecular basis for the impact of CPT on *S. frugiperda* and for future studies on potential practical application.

**Supplementary Information:**

The online version contains supplementary material available at 10.1186/s12864-021-07726-8.

## Background

The fall armyworm, *Spodoptera frugiperda* (Lepidoptera: Noctuidae), is an important insect pest worldwide. The insect can feed on at least 353 plant species including major crops such as corn, rice, soybeans, sugar cane, and cotton [1–3]. *S. frugiperda* is native to tropical and subtropical regions of the Americas, but has been spread to Africa and Asia in recent years [4]. The voracious caterpillar was first found in 2019 in Yunnan Province, China. Since then it has spread rapidly to most parts of the country [5]. Previous studies have been focused on finding control strategies such as various monitoring methods, correct identification of species and strains by genotyping, biological control and chemical application [5]. Effective insecticides for controlling *S. frugiperda* include pyrethroids, diacyl hydrazides, diamides, and benzoylureas [6]. Extensive application of insecticides caused problems including arise of populations with resistance to insecticides, toxicity to beneficial animals, and harmful effects on human health.

Plants are considered the most abundant natural resource in the world for the identification of chemicals with insecticidal activity [7, 8]. Plant secondary metabolites protect plants from herbivores and are potential candidates for novel insecticides [9]. In fact, many existing insecticides are derivatives of plant metabolites. For example, pyrethrum and nicotine are used as botanical pesticides for pest control for decades [10]. In recent years, the effect of plant secondary metabolites against *S. frugiperda* has been investigated. For example, the botanical insecticide azadirachtin is very toxic to *S. frugiperda*, with LC_50_ values 0.59 and 0.46 mg/L for 2nd and 3rd instar larvae, respectively, under 0.3 % azadirachtin emulsifiable concentrate (EC) [11]. Cedrelone, a metabolite isolated from the Australian red cedar *Toona ciliata*, is toxic to *S. frugiperda* larvae as well [12]. The flavonoid rutin extracted from soybean prolongs the development of *S. frugiperda* larvae, causing reduced larval and pupal viability [13]. The toxicity of extracts from *Actinostemon concolor*, *Piper aduncum*, and *Ruta graveolens* has also been tested against *S. frugiperda* caterpillars [14–16].

Camptothecin (CPT), a pentacyclic quinoline alkaloid isolated from the plant *Camptotheca acuminata* Decne, is a potent pharmaceutical secondary metabolite with antitumor activities in mammalian cells by targeting intracellular DNA topoisomerase I, resulting in inhibition of nucleic acid synthesis and induction of DNA strand breakage [17, 18]. CPT has also displayed insecticidal activity against several insects, including *Drosophila melanogaster*, *Musca domestica*, *Mythimna separata*, and *Spodoptera exigua* [10, 19, 20]. Field tests with 0.2 % camptothecin emulsifiable concentrate (EC) have shown high mortality on three important agricultural pests *Nilaparvata lugens* (Ståhl) *Brevicoryne brassicae* (L.), and *Chilo suppressalis* (Walker) [21]. Due to its low water solubility properties, a series of CPT derivatives have been developed through structural modification [22]. Phytophagous mites including *Tetranychus urticae*, *Acaphylla theae* and *Brevipalpus obovatus* were sensitive to the aqueous CPT-Na^+^ solution under laboratory and field conditions [23]. The toxicity mechanism indicated that CPT inhibits DNA topoisomerase I (topo I) [10]. Besides, CPT up-regulated the expression of programmed cell death protein 11 in *Spodoptera litura*, which could be involved in apoptosis induction [24]. While the effects of CPT against *S. frugiperda* and relevant molecular mechanisms remain to be revealed.

The objective of this study is to investigate the adverse effect of CPT against *S. frugiperda*. Changes in the weight of *S. frugiperda* larvae were examined after treatments with different CPT concentrations. Histopathological and ultrastructural changes in the midgut of larvae fed on diets containing 1.0 and 5.0 µg/g CPT, respectively, were examined. In addition, comparative transcriptomic analyses were carried out with different midgut samples from larvae under different treatments. Our results indicated that CPT is a growth inhibitor of *S. frugiperda* larvae and has the potential as an insecticide for controlling this important insect pest in the field.

## Results

### CPT inhibits ***S. frugiperda*** larval growth

**Fig. 1:**
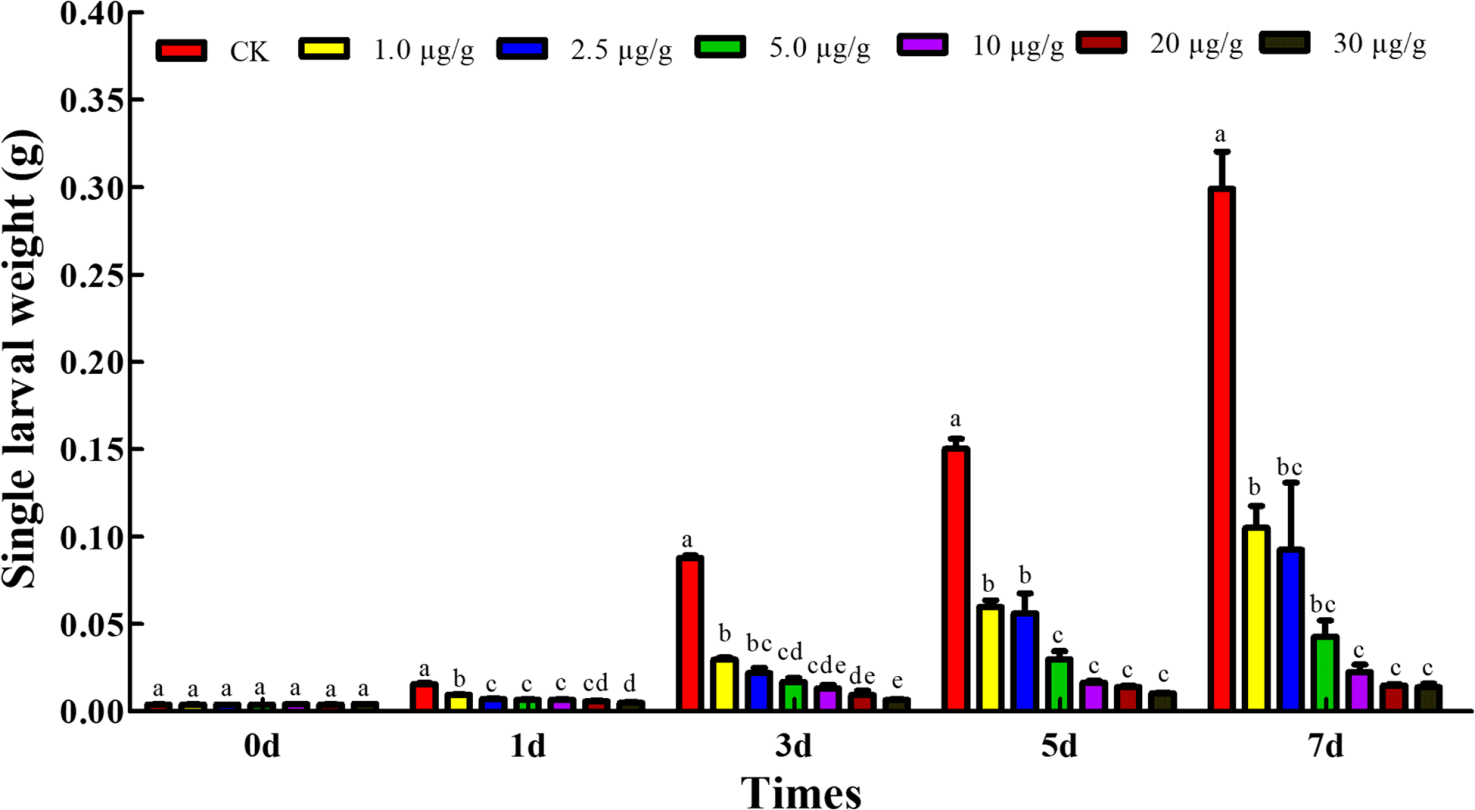
The growth inhibitory effects of CPT under different concentrations on *S. frugiperda* larvae. Average weight of individual larva was presented as mean ± SEM (n = 60). The larvae fed on the artificial diet was used as CK. Treatments were larvae fed on the diet supplemented with different concentrations of CPT. Different capital letters indicate significant differences (*p* < 0.01) between different doses as determined using ANOVA followed by DMRT

To examine any adverse effect of CPT against *S. frugiperda*, third-instar larvae were fed on artificial diets containing 0, 1.0, 2.5, 5.0, 10, 20, and 30 µg/g CPT, respectively. The weight of larvae for each sample was recorded on 1, 3, 5, and 7 days after treatments. The average weight of larvae fed on CPT-diets for one day showed no significant difference compared with that of controls. Weight loss was observed in larvae fed on CPT diets for 3, 5, and 7 days (Fig. 1). Our results indicated that CPT inhibited the growth of *S. frugiperda* larvae in a dose-dependent manner.

### CPT causes structural damages in ***S. frugiperda*** larval midgut

**Fig. 2:**
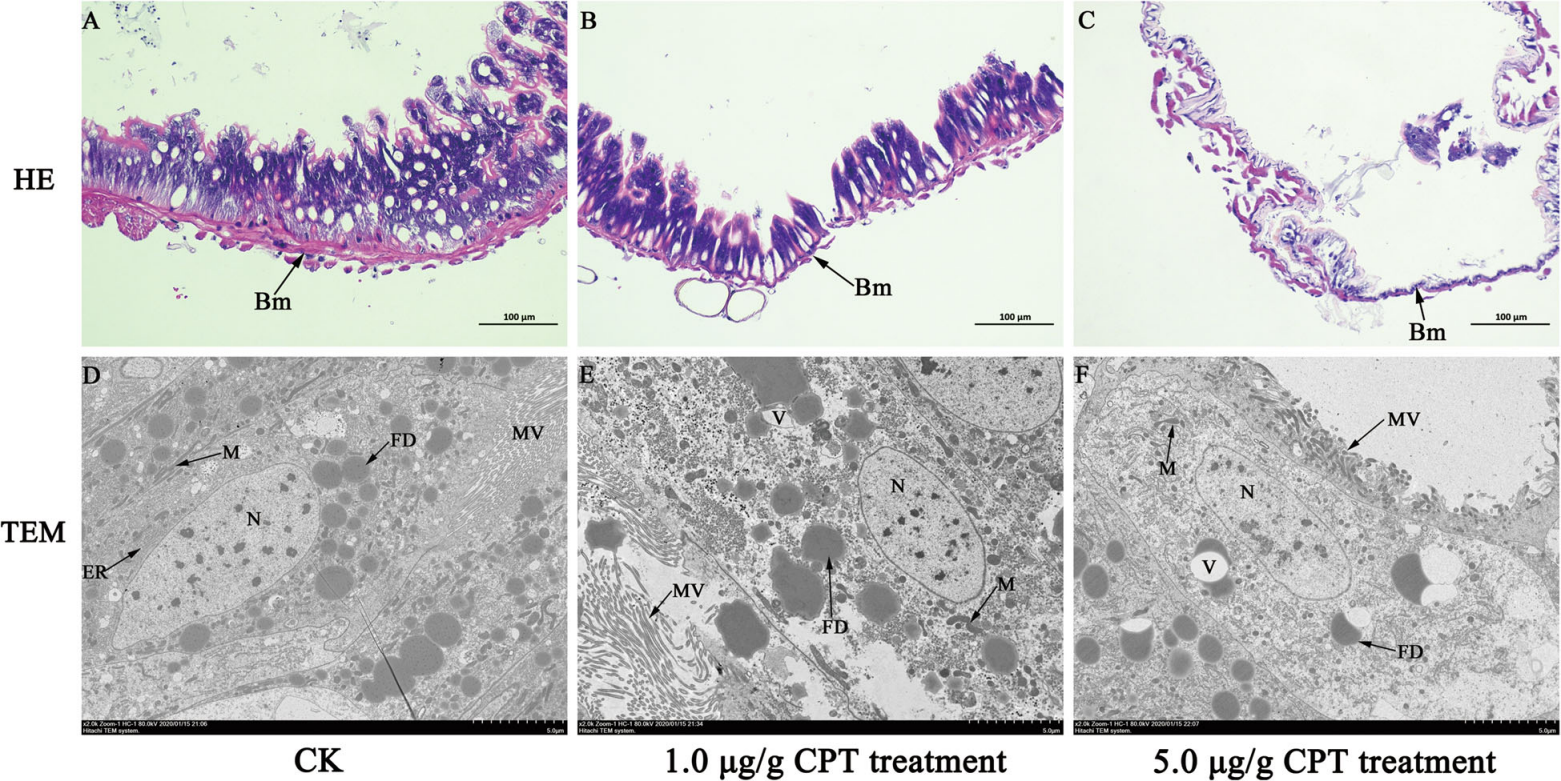
Histopathological and ultrastructural changes in the midgut of larvae fed on the diet supplemented with 1.0 and 5.0 µg/g CPT. A: Hematoxylin–eosin staining of the midgut obtained from larvae fed on a normal diet. B: Histopathological changes in the midgut dissected from larvae fed on the diet supplemented with 1.0 µg/g CPT. C: Histopathological changes of the midgut dissected from larvae fed on the diet supplemented with 5.0 µg/g CPT. D: The ultrastructure of the midgut obtained from larvae fed on normal diet. E: The ultrastructure of the midgut dissected from larvae fed on the diet supplemented with 1.0 µg/g CPT. F: The ultrastructure of the midgut dissected from larvae fed on the diet supplemented with 5.0 µg/g CPT. Me: midgut epithelium, Bm: basement membrane, L: lumen. N: nuclei, M: mitochondria, ER: endoplasmic reticulum, MV: microvilli, V: vacuole, FD: fat droplet

After 7 days of feeding, the larvae from the control group developed to sixth instar larvae, while the larvae treated with CPT grew slowly, and developed only into fourth or fifth instars. Histopathological changes were observed in the larval midgut fed on diets containing 1.0 and 5.0 µg/g CPT for 7 days based on hematoxylin-eosin (HE) staining. As shown in Fig. 2 A, midgut cells were tightly arranged in multiple layers with a thick intestinal wall in control insects. In comparison, many cells were disappeared and only a thin intestinal barrier was observed in the larval midgut fed on a 1.0 µg/g CPT diet (Fig. 2B). The severity of damage to the gut was dose-dependent. In larvae fed on a diet containing 5.0 µg/g CPT, only the basement membrane was left in the intestinal wall of the midgut, and nearly all functional cells disappeared (Fig. 2 C). Similar phenomena were observed in the gut structure under TEM. In control larvae, chromatin was evenly distributed in the nucleus. Mitochondria and endoplasmic reticulum were abundant and distributed evenly in the cytoplasm. Microvilli were ordinally distributed in the gut (Fig. 2D). In contrast, the number of mitochondria and endoplasmic reticulum decreased in midgut cells in larvae treated with 1.0 µg/g CPT. Microvilli were disorganized (Fig. 2E). In larvae fed on the 5.0 µg/g CPT diet, chromatin condensation occurred and chromatins were located close to the nuclear envelope. Microvilli decreased and deformed with large cavities (Fig. 2 F). Our results indicated that CPT had negative effects on the midgut structure of *S. frugiperda* larvae.

### Transcriptomic analyses

Midguts dissected from larvae treated with CPT (1.0 and 5.0 µg/g) for 7 days and control larvae were used for transcriptomic analyses. The number of raw reads from nine libraries ranged from 43,911,148 to 51,395,872. High quality reads ranged from 43,587,740 to 51,021,846 (Supplement Table 1). Q20 and Q30 refer to the percentage of bases with sequencing quality above 99 and 99.9 % to the total bases in the transcriptome. Values of Q20 and Q30 in each transcriptome were more than 98 and 96 %, respectively (Supplement Table 1). A total of 58,122 unigenes was obtained from the *de novo* assembling of all combined reads. The length of the unigenes ranged from 201 to 28,483 bp, with an average of 765.28 bp. N50 and GC content of the unigenes were 1358 bp and 40.55 %, respectively. Original data were deposited to the SRA database with the accession number of SRP242660. Transcripts assembled by Trinity were submitted to the TSA database with the accession number SUB8976341.

**Table 1:** The statistics of detoxification related unigenes with differentially expression in different midgut samples

Treatment	Detoxification related genes	Number of DEGs	Up-regulated	Down-regulated
CPT-1 μg/g	P450s	20	15	5
	GSTs	3	0	3
	COEs	6	5	1
	UGTs	7	5	2
	ABCs	3	0	3
	Total	39	25	14
CPT-5 μg/g	P450s	57	23	34
	GSTs	13	1	12
	COEs	8	2	6
	UGTs	11	4	7
	ABCs	19	11	8
	Total	108	41	67

### Functional annotation of unigenes

Unigenes were annotated by blasting six common databases, including NCBI non-redundant protein sequences (NR), Swiss-Prot, Protein family (Pfam), Cluster of Orthologous Groups of proteins (COG), Gene Ontology (GO), and KEGG. A total of 26,461 (45.53 %) unigenes were functionally annotated. The number of unigenes matched to NCBI NR, COG, GO, Pfam, Swiss-Prot, and KEGG databases were 24,937 (42.90 %), 22,844 (39.30 %), 18,265 (31.43 %), 16,888 (29.06 %), 16,335 (28.10 %), and 14,110 (24.28 %), respectively (Supplement Figure 1 A).

Based on BLAST results against the NR database, most (16,697 or 66.9 %) of annotated *S. frugiperda* unigenes shared the highest similarity (first hit) to sequences from *S. litura*, followed by *Helicoverpa armigera* (1447 unigenes, 5.80 %), *Trichoplusia ni* (764 unigenes, 3.06 %), *Heliothis virescens* (720 unigenes, 2.89 %), *Eumeta japonica* (388 unigenes, 1.56 %), and *C. suppressalis* (347 unigenes, 1.39 %) (Supplement Figure 1B). Only 303 unigenes (1.22 %) showed the highest similarity to sequences from *S. frugiperda*, suggesting that this important pest has been understudied genomically.

The 16,888 annotated unigenes were divided into three categories: biological process, cellular component, and molecular function. The GO terms of binding, catalytic activity, and cellular process were with the most numbers of unigenes, with 9310, 8554, and 6308 in each category, respectively (Supplement Figure 1 C). The unigenes with KEGG annotations could be classified into five major categories, including Metabolism (3182 unigenes), Genetic Information Processing (2507 unigenes), Environmental Information Processing (1889 unigenes), Cellular Processes (1987 unigenes), and Organismal Systems (2663 unigenes) (Supplement Figure 1D). For the secondary categories, the pathways of signal transduction, translation, and carbohydrate metabolism were ranked as the top three subcategories, with 1681, 1200, and 1079 unigenes, respectively, in each subcategory.

### Identification of DEGs based on transcriptomes

**Fig. 3:**
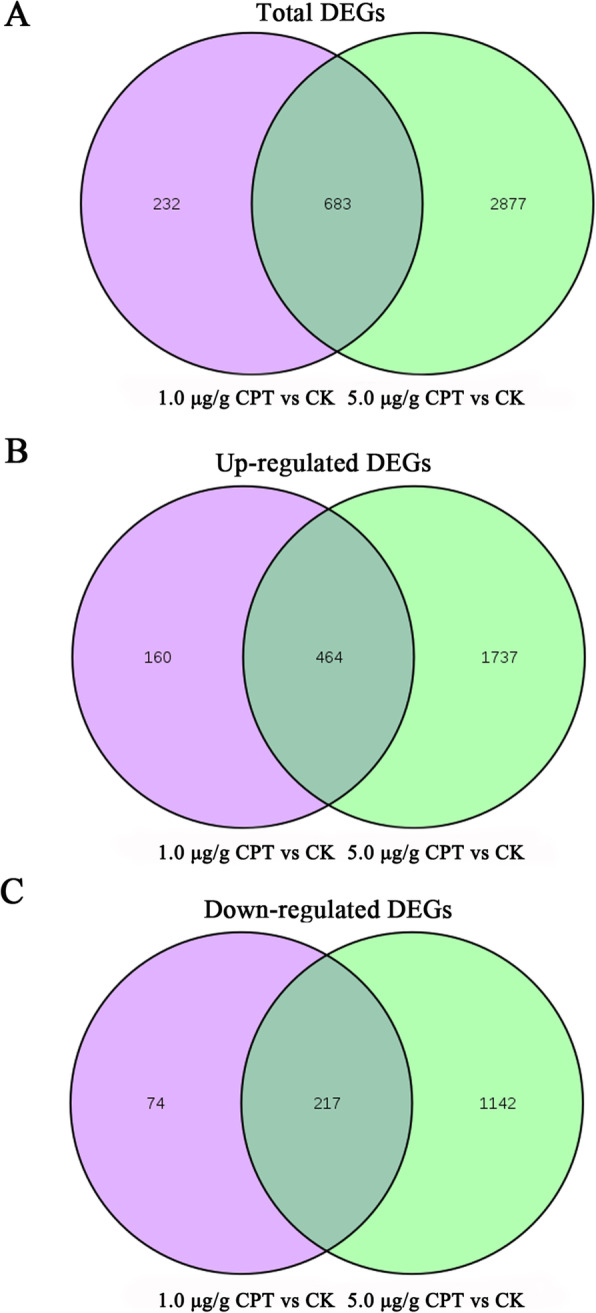
A venn diagram of DEGs obtained from different comparative analyses. A: A venn diagram of total DEGs obtained from different comparative analyses. There were 683 unigenes that exhibited differential expression between samples treated with 1.0 and 5.0 µg/g CPT. B: A venn diagram of up-regulated DEGs obtained from different comparative analyses. There were 464 unigenes that exhibited up-regulated expressions in samples treated with 1.0 and 5.0 µg/g CPT when compared to control. C: A venn diagram of down-regulated DEGs obtained from different comparative analyses. There were 217 unigenes that exhibited down-regulated expressions in samples treated with 1.0 and 5.0 µg/g CPT when compared to control. Purple ring represents DEGs identified from the comparison between controls and samples treated with 1.0 µg/g CPT. Green ring represents DEGs identified from the comparison between controls and samples treated with 5.0 µg/g CPT

A total of 915 unigenes were expressed differentially between controls and samples treated with 1.0 µg/g CPT. Compared to the control group, 612 unigenes were up-regulated and 291 unigenes were down-regulated in the group treated with 1.0 µg/g CPT (Fig. 3 A). The number of DEGs between control and 5.0 µg/g CPT-treated samples increased to 3560. Among the DEGs, 2201 were up-regulated and 1359 down-regulated (Fig. 3 A). Comparative analyses revealed that 683 unigenes were differentially expressed in both 1.0 and 5.0 µg/g CPT-treated samples when compared to control. Among the common DEGs, 464 were up-regulated and 217 down-regulated (Fig. 3B and C).

A large number of DEGs were genes involved in detoxification, including genes encoding cytochrome P450 monooxygenases (P450s), glutathione S-transferases (GSTs), carboxylesterases (COEs), UDP glucosyltransferases (UGTs), and ATP-binding cassette transporters (ABCs). As shown in Table 1 and 39 detoxification genes were differentially expressed between controls and samples treated with 1.0 µg/g CPT. These differentially expressed genes encode 20 P450s, 3 GSTs, 6 COEs, 7 UGTs, and 3 ABCs. Most of these DEGs were up-regulated, including 15 coding for P450s, 5 for COEs, and 5 for UGTs (Table 1). The number of detoxification genes expressed differentially between controls and samples treated with 5.0 µg/g CPT increased to 108, including genes encoding 57 P450s, 13 GSTs, 8 COEs, 11 UGTs, and 19 ABCs. Among the up-regulated DEGs, 23 were genes coding for P450s, 1 for GST, 2 for COEs, 4 for UGTs, and 11 for ABCs (Table 1).

**Fig. 4:**
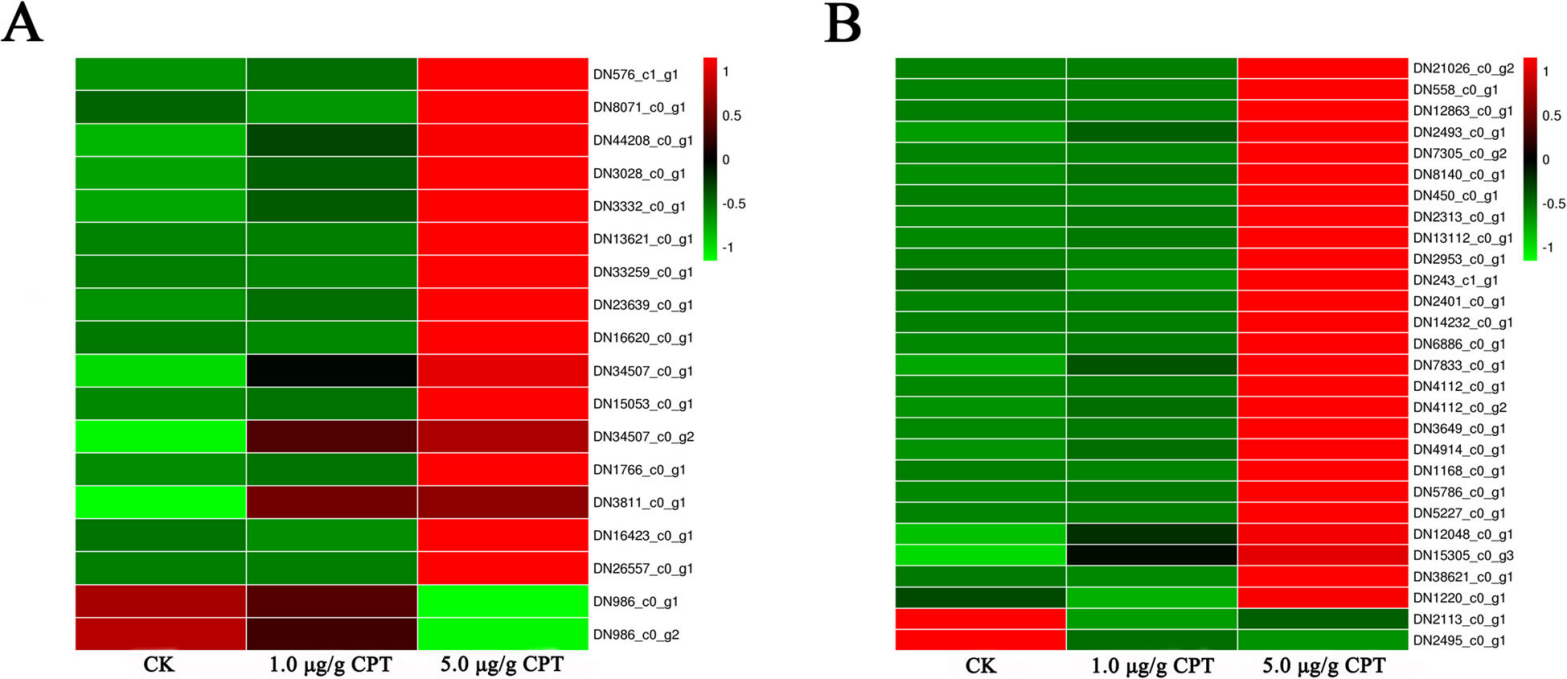
Heatmaps of selected DEGs in response to CPT treatments. A: The heatmap of differentially expressed unigenes encoding mucins after CPT treatments. B: The heatmap of differentially expressed unigenes coding for cuticle proteins

In addition to DEGs with functions in detoxification, several genes encoding mucins were also expressed differentially among control and treated samples. Mucins are high molecular weight glycoproteins covering the surface of epithelial cells that respond to external environmental stimuli such as infection, dehydration, and physical and chemical injury [25]. Two and 18 genes encoding mucins were differentially expressed between controls and samples treated with 1.0 and 5.0 µg/g CPT, respectively. The unigenes encoding mucin-5AC (DN34507_c0_g1) and mucin-17 (DN3811_c0_g1) were up-regulated in samples treated with 1.0 µg/g CPT when compared to control. Most (16) DEGs encoding mucin proteins were up-regulated in samples treated with 5.0 µg/g CPT, and only two mucin genes were down-regulated (Fig. 4 A).

The third major group of DEGs included genes encoding cuticle proteins (CPs), which are indispensable structural components for insect tissues such as cuticle and midgut peritrophic membrane. Specifically, Four genes encoding larval cuticle protein LCP-17 (DN2495_c0_g1), cuticle protein 6.4-like (DN2113_c0_g1), cuticle protein CP14.6-like (DN38621_c0_g1) and cuticular protein RR-2 (DN1220_c0_g1) were down-regulated in samples treated with 1.0 µg/g CPT. Interestingly, 26 unigenes encoding cuticle proteins were up-regulated in samples treated with 5.0 µg/g CPT, whereas there were only two cuticle protein-encoding genes that were down-regulated (Fig. 4B).

### GO and KEGG analyses

A total of 553 DEGs between controls and samples treated with 1.0 µg/g CPT were assigned to 175 GO terms. Among these GO terms, 30 were enriched significantly (corrected *P*-values < 0.05). The enriched GO terms for biological process included “carbohydrate derivative metabolic process”, “aminoglycan metabolic process”, “chitin metabolic process”, “glucosamine-containing compound metabolic process”, and “amino sugar metabolic process”. The enriched GO terms for cell component included “membrance part”, “integral component of membrane” and “intrinsic component of membrane”. The enriched GO terms for molecular function included “oxidoreductase activity”, “transporter activity”, and “transmembrane transporter activity” (Supplement Figure 2 A).

A total of 2000 DEGs between controls and samples treated with 5.0 µg/g CPT were assigned to 215 GO terms. Among these GO terms, 43 were enriched significantly (corrected *P*-values < 0.05). The most significantly enriched GO term for biological process was “chitin metabolic process” (corrected *P*-value = 7.27901E-08, 50 DEGs). The most significantly enriched GO term for cellular component was “extracellular region” (corrected *P*-value = 8.61215E-08, 107 DEGs). The most significantly enriched GO term for molecular function was “structural constituent of cuticle” (corrected *P*-value = 7.27901E-08, 35 DEGs) (Supplement Figure 2B).

**Fig. 5:**
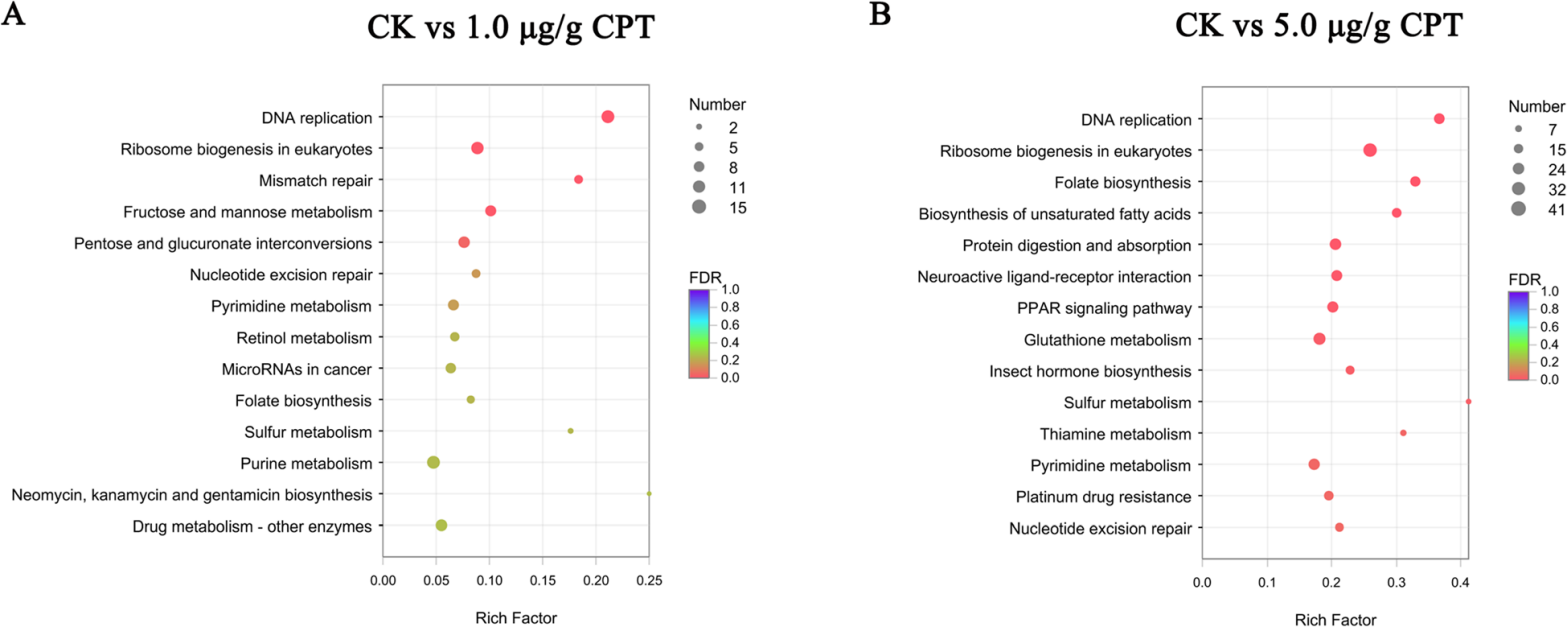
KEGG pathway analyses of identified DEGs. A: The top 14 pathways enriched with DEGs obtained from midgut samples from larvae treated with 1.0 µg/g CPT with FDR values. Among them, five pathways were significantly enriched with corrected *P*-values < 0.05. B: The 14 pathways significantly enriched with DEGs obtained from midgut samples from larvae treated with 5.0 µg/g CPT (corrected *P*-values < 0.05). The x-axis represents rich factor

KEGG analysis revealed that 369 DEGs between controls and samples treated with 1.0 µg/g CPT were assigned to 178 pathways. Among the 178 pathways, five were enriched significantly (corrected *P*-values < 0.05), including “DNA replication” (15 DEGs), “Purine metabolism” (15 DEGs), and “Ribosome biogenesis in eukaryotes” (14 DEGs). KEGG analysis assigned 1401 DEGs between controls and samples treated with 5.0 µg/g CPT to 232 pathways. The most significantly enriched pathways included “Purine metabolism” (42 DEGs), “Ribosome biogenesis in eukaryotes” (41 DEGs), and “Peroxisome” (36 DEGs) (Fig. 5 C and 5D). “DNA replication” was the most significantly enriched KEGG pathway in both samples treated with either 1.0 or 5.0 µg/g CPT.

### qRT-PCR validation

**Fig. 6:**
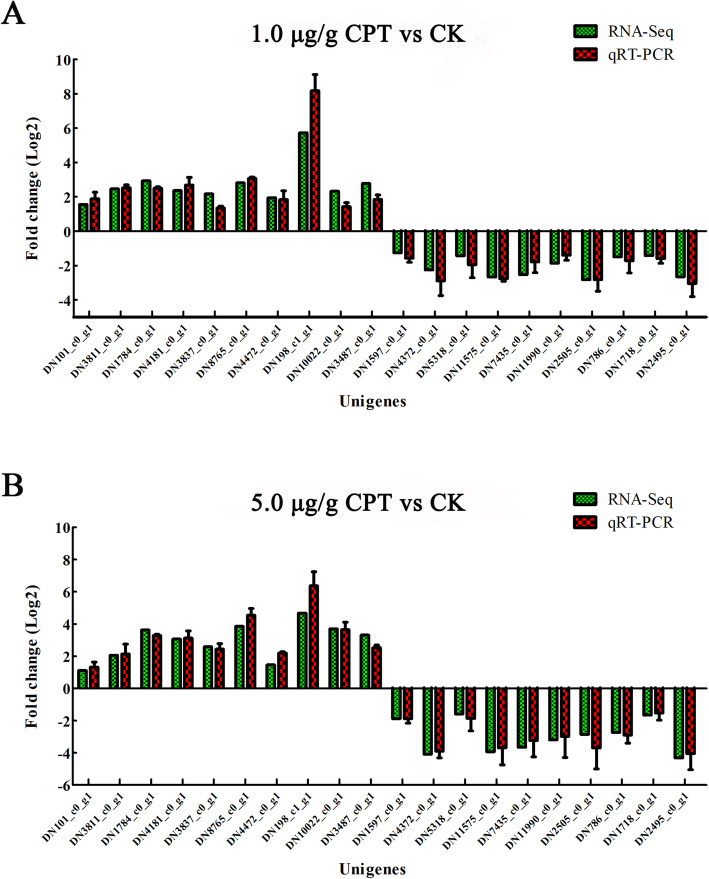
Quantitative real-time PCR (qRT-PCR) and RNA-Seq data of selected genes. Ten DEGs up-regulated and 10 DEGs down-regulated were selected for PCR analysis. RPL3 and RPL13 were used as the reference genes for qRT-PCR normalization. mRNA expression levels of the selected genes were calculated using the 2^−△△CT^ method

To confirm the results of transcriptomic analyses, 20 unigenes including genes involved in detoxification and DNA replication, genes encoding mucins and cuticle proteins genes, were selected for qRT-PCR validation. As shown in Fig. 6, the expression patterns of the selected genes in *S. frugiperda* midguts changed significantly after CPT treatments based on qRT-PCR analysis. The changes in gene expression levels based on qRT-PCR were largely consistent with the transcriptomic data.

## Discussion

*S. frugiperda* has become a serious insect pest in China in the past couple of years [26]. Various chemicals such as chlorantraniliprole, spinetoram, emamectin benzoate, spinetoram, acephate, and pyraquinil have been evaluated to control this pest in the field [27–29]. Some bioactive compounds including azadirachtin isolated from *Azadirachta indica* and celangulins extracted from the medicinal plant *Celastrus angulatus* have been studied for potential control of this destructive insect [11, 30]. CPT is a natural indole alkaloid used for cancer therapy [31]. The insecticidal activity of CPT against other insect pests has also been investigated. In this study, development delay was induced in *S. frugiperda* larvae treated with CPT, but no mortality was observed. This result may be due to the high number of detoxifying enzyme genes that are often in polyphagous pests [32]. In addition, synergism between CPT and *Bacillus thuringiensis* (*Bt*) or nucleopolyhedroviruses exists against *Trichoplusia ni* and *S. exigua* [33]. Our results showed that CPT can inhibit the growth of *S. frugiperda* larvae. Therefore, CPT might be used as an independent insecticide for controlling *S. frugiperda*. Alternatively, CPT may be used with other insecticides for enhanced efficiency. One limitation of CPT as an insecticide is its insolubility in water. More efficient derivatives with improved solubility and hydrophobicity may be developed in the future for pest control.

The insect midgut is an important organ responsible for food digestion and nutrient absorption [34, 35]. CPT has been reported to induce alterations in the midguts of *Trichoplusia ni* and *S. exigua* larvae, including the loss of the single layer of epithelial cells and the disruption of the peritrophic membrane [33]. In this study, we observed the loss of epithelial cells, abnormal cell structure, and intestinal wall degradation in the midgut of *S. frugiperda* after CPT treatments. These observations are consistent with previous findings in other insects, suggesting that CPT holds the potential as an insecticide for controlling *S. frugiperda* and other insect pests.

Recently, transcriptomic analysis has become a routine method to identify the differentially expressed genes in insects in response to toxic compounds [36]. For example, transcriptomic analyses have been carried out to identify DEGs in the Chinese populations of *S. frugiperda* in response to 23 pesticides [37, 38]. DEGs in *S. frugiperda* larvae treated with azadirachtin were also initially analyzed [36]. In this study, DEGs in *S. frugiperda* larvae treated with CPT were analyzed for the first time. Our transcriptomic analyses of midguts from *S. frugiperda* larvae revealed that the expression levels of a large number of genes were affected by CPT treatments.

Among the up-regulated genes by CPT are genes involved in detoxification. Metabolic detoxification through the overexpression of metabolic genes is considered one of the main ways to handle toxic insecticides by pests [39]. The insect midgut is an important tissue responsible for pesticide detoxification where a variety of detoxification enzymes are produced [40]. Insect midgut often increases the expression of metabolic genes in response to pesticide treatments. For example, the transcription levels of detoxifying-related genes including P450s and GSTs were up-regulated by low-dose of acetamiprid in the midgut of *B. mori* [40]. Sublethal concentrations of Cry1Ca protein altered the expressions of P450s, CarEs, and GSTs in *S. exigua* larval midgut [41]. Detoxification-related genes including those encoding P450s, GSTs, and COEs are up-regulated in *S. litura* larval midguts after being treated with tomatine [42]. The roles of several detoxification genes in pesticide resistance in insects have been validated by RNA interference, including CYP321A8, CYP321A9, and CYP321B1 in *S. frugiperda*, a GST in *Ostrinia furnacalis*, Pxae18 and Pxae28 in *Plutella xylostella* [43–45]. Here we found that a large number of detoxification-related genes were coordinately up-regulated in the midgut of *S. frugiperda* larvae after CPT treatments. We speculated that up-regulation of these detoxification genes such as the genes encoding P450s, GSTs, and CarEs could accelerate the removal of CPT, thus reducing the toxicity of this chemical to *S. frugiperda*. Our results indicated that high doses of CPT induced more detoxification-related genes. The dose-dependent upregulation of these genes further indicates their roles in the detoxification of CPTs in *S. frugiperda* larvae. Assuming the up-regulated detoxification genes are indeed critical to detoxify CPT, application of CPT together with detoxification enzyme inhibitor(s) might increase the efficacy of CPT as a controlling agent for the *S. frugiperda* pest.

ABC transporters are membrane proteins that are divided into eight subfamilies (A to H) based on the conserved nucleotide-binding domain (NBD) and their functions [46]. Three ABCG subfamily genes are up-regulated in *H. armigera* after being treated with abamectin, indoxacarb, and lambda-cyhalothrin [47]. Five ABC transporters are induced in *B. mori* treated with NaF [46]. In the present study, eleven ABC transporters were up-regulated in *S. frugiperda* larvae treated with 5.0 µg/g CPT. These up-regulated genes belong to either ABCC or ABCG subfamilies. The ABCC transporters in most insects are composed of full transporters (FTs) and half transporters (HTs), while ABCG transporters are typical HTs, which need to form homo- or heterodimers to perform transport functions [48]. There is evidence to suggest that ABC transporters from the ABCC and ABCG subfamilies are involved in insecticide resistance [49, 50]. Therefore, our results may suggest that these up-regulated ABC transporters might be involved in CPT metabolism in *S. frugiperda*.

UGTs perform diverse functions including substrate detoxification, ecdysteroid metabolism, cuticle formation, pigmentation, and olfaction in insects. Lipophilic compounds could conjugate with UDP-glucose of UGTs and be glycosylated as water-soluble products that can be easily excreted [51]. Multiple UGTs were found involved in xenobiotics detoxification and insecticide resistance. Several UGTs are up-regulated in nymphs of *N. lugens* exposed to β-asarone [52]. Ten UGTs are differentially expressed in harmine-treated Sf9 cells [53]. In this study, five and four UGTs were identified as DEGs in *S. frugiperda* larvae treated with 1.0 and 5.0 µg/g CPT, respectively. Two UGT genes were previously found involved in the detoxification of benzoxazinoids from maize in *S. frugiperda* larvae [54]. These findings indicated that UGTs could play roles in CPT detoxification.

Mucins are highly O-glycosylated proteins that are abundant in salivary glands, the midgut, and malpighian tubules of insects [55]. Mucins play an important role in lubricating the epithelial cells and protecting tissues from physical and chemical injuries [56]. Mucins are also involved in host adaptation and oviposition regulation [56, 57]. In *S. frugiperda*, mucins are located in the peritrophic membrane and thus may protect epithelial cells and immobilize proteolytic enzymes [57]. Multiple genes encoding different mucins were mostly up-regulated in samples treated with CPT, suggesting that mucins may have participated in repairing damages caused by CPT in the peritrophic membrane as reported previously [58, 59]. In addition to mucins, cuticle proteins are also primary components of the peritrophic membrane [60]. Several genes encoding different cuticle proteins were also induced by CPT treatments. The up-regulation of genes encoding both mucins and cuticle proteins suggested that the peritrophic membrane in the midgut of *S. frugiperda* larvae may have gained increased resistance in this insect to CPT. If that is the case, application of CPT with other compounds that can prevent induction of mucin or cuticle protein genes or both may result in enhanced efficiency of CPT in controlling this insect pest. Furthermore, the expressions of DNA topoisomerases were not changed after CPT treatments. The likely reason is that CPT could affect the activities of topoisomerases, but does not affect the transcription level of topoisomerases.

Carbohydrates, lipids, proteins, nucleotides, chitin, and vitamins are important resources of energy for insect growth and development [35]. Our GO analyses revealed that many metabolic processes were enriched with DEGs significantly, including chitin metabolic process, glucosamine-containing compound metabolic process, aminoglycan metabolic process, amino sugar metabolic process, and carbohydrate derivative metabolic process (corrected *P*-values < 0.05). Innate immune response and immune system processes were enriched (corrected *P*-values < 0.05) as well. KEGG analyses demonstrated that some pathways associated with Carbohydrate metabolism, Amino acid metabolism, Lipid metabolism, Nucleotide metabolism, Xenobiotics biodegradation, and Metabolism of cofactors and vitamins were enriched significantly, as well as the pathways of Digestive system, Endocrine system, Cell growth and death (corrected *P*-values < 0.05). These results suggest that CPT inhibited the growth of *S. frugiperda* larvae by disrupting many digestive and metabolic processes.

## Conclusions

In conclusion, growth inhibition was observed in *S. frugiperda* larvae treated with different concentrations of CPT. Damage in the midgut was found after CPT treatments based on HE staining and TEM observation. Comparative transcriptomic analyses identified a large number of DEGs caused by CPT treatments. Genes involved in detoxification, DNA replication, and structural components of the peritrophic membrane were up-regulated significantly. Our results suggest that CPT exerted its impact on the growth of *S. frugiperda* larvae potentially by disrupting DNA replication, the digestive system, and other metabolic processes. Our studies provided a foundation for future research on the basic mechanism and potential practical application of CPT on *S. frugiperda* control.

## Methods

### Insect rearing

A laboratory colony of *S. frugiperda* was generated from larvae collected from a cornfield in Conghua District, Guangzhou City, Guangdong Province, China. The colony has been maintained on an artificial diet since then. The artificial diet of 1 kg was prepared with 100 g cornflour, 80 g soy flour, 26 g yeast powder, 26 g agar, 8.0 g vitamin C, 2.0 g sorbic acid, 1.0 g choline chloride, 0.2 g inositol, 0.2 g cholesterol, and 900 mL distilled water. Adults were fed on 10 % honey water. Insect cultures were kept in an incubator set at 25 ± 1 °C, 60–70 % relative humidity, and a 16:8 h light: dark cycle.

### Treatments

CPT was purchased from Selleck Chemicals (USA) and was dissolved in dimethyl sulfoxide (DMSO). Third-instar larvae were selected and fed on artificial diets containing CPT at 1.0, 2.5, 5.0, 10, 20, and 30 µg/g, respectively, for 7 days. Twenty larvae were selected as one sample for the experiment and three biological replicates of each concentration were performed. Weights of insects for each sample were recorded after feeding for 1, 3, 5, and 7 days, respectively.

### Hematoxylin–eosin staining

After seven days of feeding, the midgut of *S. frugiperda* larvae treated with 1.0 and 5.0 µg/g CPT was dissected and washed with cold phosphate-buffered saline (PBS). Midgut tissues were fixed with 4 % paraformaldehyde (#G1101, Servicebio, Wuhan, China) at 4 °C for more than 24 h. Midgut samples were then embedded into paraffin wax and sliced into sections of 4 μm thickness. The sections were fixed onto a glass slide and stained with hematoxylin and eosin solution. Histopathological changes in the midgut of larvae fed on CPT were visualized on a microscope (Nikon, Japan).

### Transmission electron microscope observation

Larval midguts exposed to 1.0 and 5.0 µg/g CPT for 7 days were dissected, washed with cold PBS, and then fixed in glutaraldehyde solution at 4 °C for 4 h. After washing with PBS three times, the tissues were postfixed in 1 % osmic acid solution at room temperature for 2 h and then washed with PBS three times again. The tissues were dehydrated in a graded series of ethanol solutions (50–100 %) and then kept in 100 % acetone. The midguts were then embedded in epoxy resin and sliced into sections of 60–80 nm thickness. After staining with 2 % uranium acetate saturated alcohol solution and 2 % lead citrate solution for 15 min each time, the sections were dried at room temperature overnight. The ultrastructures of larval midgut were observed on an HT7800 TEM (HITACHI, Japan).

### RNA isolation and Illumina sequencing

Ten to fifteen larval midgut samples exposed to 1.0 and 5.0 µg/g CPT for 7 days were collected, Total RNA was extracted using TRIzol® Reagent (Invitrogen, USA). Potential genomic DNA contaminants were removed using DNase I (TAKARA, Japan). The quality and concentration of RNA samples were analyzed on a 2100 Bioanalyser (Agilent, USA) and quantified on an ND-2000 (NanoDrop Technologies, USA). cDNA library construction and RNA-seq were conducted at Shanghai Majorbio Bio-pharm Biotechnology Co., Ltd. (Shanghai, China) via a commercial contract. Briefly, mRNA was purified from 5 µg total RNA of each sample using oligo-dT-attached magnetic beads. Purified mRNA was fragmented using a fragmentation buffer and the resulting products were used as templates for double-stranded cDNA synthesis with a SuperScript double-stranded cDNA synthesis kit (Invitrogen, CA). The double-stranded cDNA was subjected to end-repair, phosphorylation, and ‘A’ base addition. cDNA fragments of 200–300 bp were selected by 2 % Low Range Ultra Agarose and amplified in PCR reaction with Phusion DNA polymerase (New England Biolabs, Boston, MA) for 15 cycles. Then the products were quantified on TBS380 and sequenced on the Illumina HiSeq 4000 platform (Illumina, USA).

### De novo assembly and annotation

After sequencing, the base quality, base error rate, and A/T/G/C base content distribution statistics of raw paired-end reads from each sample were evaluated with the software of fastx_toolkit_0.0.14 (http://hannonlab.cshl.edu/fastx_toolkit/). The reads that contained adaptor contamination, low-quality bases, and undetermined bases in raw reads were removed with SeqPrep (https://github.com/jstjohn/SeqPrep) and Sickle (https://github.com/najoshi/sickle) under the default parameters. The clean reads were assembled using Trinity (https://github.com/trinityrnaseq/trinityrnaseq) with the parameters of k-mer = 25 and min_kmer_cov = 5. Then the false assembled transcripts include chimera, structural errors, incomplete assembly, base errors, etc. were filtered with TransRate (http://hibberdlab.com/transrate/). Besides, the redundant sequences were identified and removed by CD-HIT (http://weizhongli-lab.org/cd-hit/). Subsequently, the assembly integrity of these transcriptomes was assessed by BUSCO (Benchmarking Universal Single-Copy Orthologs, http://busco.ezlab.org). The transcripts that shared sequence content were clustered and the longest transcript in the cluster was selected as the unigene. Assembled transcripts and unigenes were annotated by searching the NCBI protein non-redundant (NR), Swiss-Port, and Clusters of Orthologous Groups of proteins (COG) databases with DIAMOND v0.8.37.99 software using BLASTX with a threshold E-value of 1 × 10^− 5^. Besides, the Pfam database was also used for unigenes and transcripts annotation with the software of HMMER3 3.1b2. The function of each transcript was assigned according to the first hit. Gene Ontology (GO) annotation and Kyoto Encyclopedia of Genes and Genomes (KEGG) pathway analyses were performed using BLAST2GO 2.5.0 (http://www.blast2go.com/b2ghome) and KOBAS 2.1.1 (KEGG, http://www.genome.jp/kegg/) with default parameters, respectively.

### Differentially expressed genes (DEGs)

The method of fragments per kilobase of exon per million mapped reads (FRKM) was used to analyze the expression level of each transcript in different samples. Differentially expressed genes (DEGs) were identified using R statistical package software named EdgeR (Empirical Analysis of Digital Gene Expression in R, http://www.bioconductor.org/packages/2.12/bioc/html/edgeR.html). The criteria for DEGs were |log2Fold change| ≥1 and P-value ≤ 0.05. GO functional enrichment analysis of DEGs was performed via Goatools (https://github.com/tanghaibao/Goatools) with Fisher’s exact test. KEGG pathway enrichment analysis was conducted by KOBAS software (http://kobas.cbi.pku.edu.cn/home.do) with Fisher’s exact test and the pathway enriched with DEGs significantly was identified with the judging indicator of corrected *P*-value < 0.05.

### qRT-PCR

Two µg total RNA of each sample was used for qRT-PCR cDNA synthesis using a FastKing RT Kit (With gDNase) (KR116, TIANGEN, Beijing, China) following the manufacturer’s instruction. Primers were designed using the Primer 5.0 software and synthesized by Thermo Scientific. Primer sequences were listed in Supplement Table 2. The *TransStart*® Tip Green qPCR SuperMix (AQ141, TransGen Biotech, Beijing, China) was used for qRT-PCR in a 10 µL reaction solution on a LighCycler480 II machine (Roche, Basel, Switzerland). qRT-PCR has proceeded as follows: one cycle of denaturation at 94 °C for 3 min, followed with 45 cycles of denaturation at 94 °C for 15 s, annealing at 60 °C for 20 s, and elongation at 72 °C for 20 s, followed by a melting curve analysis. Two reference genes RPL3 (AY072287.1) and RPL13 (AF400183.1) were selected for normalization of qRT-PCR results. mRNA levels were analyzed by the 2^−△△CT^ method. Each assay was repeated three times.

### Data analysis

Each experiment was conducted with three biological replicates and all data were expressed as the mean ± standard deviation (SD). One-way ANOVA was applied for statistical analysis of single larval weight. Homogeneity of variance test was determined by *F* test and different capital letters in figures indicate significant differences between different doses as determined using ANOVA followed by Duncan’s new multiple range method (DMRT) (*p* < 0.01) in SPSS 17.0 (IBM, USA).

## Supplementary Information

**Additional file 1:**

## Data Availability

The raw reads of transcriptomes in this study have been deposited in the NCBI SRA database with the accession number of SRP242660.

## Abbreviations

CPT: : Camptothecin
DEGs: Differentially expressed genes
KEGG: Kyoto Encyclopedia of Genes and Genomes
EC: emulsifiable concentrate
Topo I: DNA topoisomerase I
Pfam: Protein family
COG: Cluster of Orthologous Groups of proteins
NR: NCBI protein nonredundant
GO: Gene Ontology
P450s: Cytochrome P450 monooxygenases
GSTs: Glutathione S-transferases
COEs: Carboxylesterases
UGTs: UDP glucosyltransferases
ABCs: ATP-binding cassette transporters
CPs: Cuticle proteins
*Bt*: *Bacillus thuringiensis*
DMSO: Dimethyl sulfoxide
HE: Hematoxylin–eosin
PBS: Phosphate-buffered saline
RNA-seq: RNA-sequencing
FRKM: Fragments per kilobase of exon per million mapped reads
qRT-PCR: quantitative real time polymerase chain reaction
RPL3: Ribosomal Protein L3
SD: Standard deviation
DMRT: Duncan’s new multiple range method
