# Supplementary material for: Growth inhibition of Spodoptera frugiperda larvae by camptothecin correlates with alteration of the structures and gene expression profiles of the midgut
Source: BMC Genomics. 2021 May 26;22:391. doi: 10.1186/s12864-021-07726-8 (PMC8157707; doi:10.1186/s12864-021-07726-8)
Supplement: Supplementary file 1 — Additional file 1: [file 12864_2021_7726_MOESM1_ESM.docx]

**Camptothecin inhibited the growth of *Spodoptera frugiperda* larvae by altering the structures and gene expression profiles of midgut**

Benshui Shu, Yan Zou, Haikuo Yu, Wanying Zhang, Xiangli Li, Liang Cao, Jintian Lin*

Guangzhou City Key Laboratory of Subtropical Fruit Trees Outbreak Control, Zhongkai University of Agriculture and Engineering, Guangzhou, China

* Correspondence authors at: Guangzhou City Key Laboratory of Subtropical Fruit Trees Outbreak Control, Institute for Management of Invasive Alien Species, 313 Yingdong teaching building, Zhongkai University of Agriculture and Engineering, Guangzhou, 510225, PR China.

E-mail addersses: [linjtian@163.com](mailto:linjtian@163.com) (J Lin).

Supplement Table 1 Summary of the transcriptome sequencing data

| Sample name | Raw reads | Clean reads | Error rate (%) | Q20 (%) | Q30 (%) | GC content (%) |
| --- | --- | --- | --- | --- | --- | --- |
| CK1  CK2  CK3  1.0 μg/g CPT-1  1.0 μg/g CPT-2  1.0 μg/g CPT-3  5.0 μg/g CPT-1  5.0 μg/g CPT-2  5.0 μg/g CPT-3 | 51,083,926  50,930,686  50,287,542  51,395,872  43,911,148  51,402,050  44,557,234  48,036,842  49,530,172 | 50,726,216  50,587,806  49,965,332  51,006,978  43,587,740  51,021,846  44,211,534  47,695,420  49,192,174 | 0.023  0.023  0.0229  0.0229  0.0233  0.0231  0.0231  0.0231  0.0231 | 98.85  98.85  98.9  98.89  98.73  98.8  98.82  98.83  98.85 | 96.17  96.17  96.25  96.27  95.84  96.05  96.01  96.1  96.11 | 49.58  49.93  49.17  49.81  49.59  50.22  47.03  49.45  47.97 |

Supplement Table 2. Primers used for RT-qPCR in the paper

| Primer name | Primer Sequence (5’-3’) | Primer name | Primer Sequence (5’-3’) |
| --- | --- | --- | --- |
| DN101_c0_g1-F  DN101_c0_g1-R  DN3811_c0_g1-F  DN3811_c0_g1-R  DN1784_c0_g1-F  DN1784_c0_g1-R  DN4181_c0_g1-F  DN4181_c0_g1-R  DN3837_c0_g1-F  DN3837_c0_g1-R  DN8765_c0_g1-F  DN8765_c0_g1-R  DN4472_c0_g1-F  DN4472_c0_g1-R  DN198_c1_g1-F  DN198_c1_g1-R  DN10022_c0_g1-F  DN10022_c0_g1-R  DN3487_c0_g1-F  DN3487_c0_g1-R  RPL3-F  RPL3-R | TGTCCTTACATCTGGTCGTCG  CCACAGTTCACTAACTCGCTCC  AGCAAACTGCCGAAGAAAAT  GTTGTAAAACTGCTTGGCGATA  ACACTGGACGCCGCTATGAC  GATTGTTTGTTCCGAGACTTGAGA  CTGAGCTAGTCTTGGGCACG  TGGCACATACTGGGTTACGG  GTGGACAGCATGGTCGAAGTG  TTGGTGACAGCGGTGGGTT  AGTTGTTCCAGCAGAGTCAATG  GCCTGTAGGGTCTTCCAGAT  TTTGGAGCCGTTCCCTTTT  CATTTCAATGAGTGGCAGCATA  GGAGTTGAGACCCAGACGATG  CATTCCTGGATGTCGGATTGT  CTGAGGAACCCCGATGTGA  GGTGAATGCGTTGTTGCTAG  GCTCACCAAGAGGAAGAACCC  GCAGTGGACAGTCGCAGTATG  AAGCCAGTCCACCTTACCGC  GCCCAAACAGTGAGCAGAGC | DN1597_c0_g1-F  DN1597_c0_g1-R  DN4372_c0_g1-F  DN4372_c0_g1-R  DN5318_c0_g1-F  DN5318_c0_g1-R  DN11575_c0_g1-F  DN11575_c0_g1-R  DN7435_c0_g1-F  DN7435_c0_g1-R  DN11990_c0_g1-F  DN11990_c0_g1-R  DN2505_c0_g1-F  DN2505_c0_g1-R  DN786_c0_g1-F  DN786_c0_g1-R  DN1718_c0_g1-F  DN1718_c0_g1-R  DN2495_c0_g1-F  DN2495_c0_g1-R  RPL13-R  RPL13-F | TCGCTGGTTTCGGAGTCAC  TTAGGAATGTACGGGGATGTTT  ACCTCTGGTTGCCATCCCT  TCTTCCACAGTCAACTTCTCCAT  AGAGTGCGTGACAACTTCGTG  ATGTTGCCCCATAGACCGA  TGACAAACGCTGTTGTTAGGA  CGTGCTGGATTGAGTATGGA  GCTGGTGAGACCTATGCTAGACA  GGAATGGTGGTGATGATGCC  AGCGGGTATGGACGTACTGAC  CAAGGGTTCTGCCTGGTGTC  AATGCGGGGCTGAAGGAT  TTCCAACACCACCAATGACG  TGAAGAAGAACCCACAACATACAG  AGCCTTGAGTTGATGGTCGC  AGTGCAGCAGTAGATATTCAGCC  TGTTTTATTCGTCCTCGGGTT  CTGGTGTCCTCAAGAACGCTAA  GGCTTCCCTGGGGTCTGTAT  GCTTCGCCCTTCAATACCTTC  GCCTTAACCCTGCTTTTGCTAG |


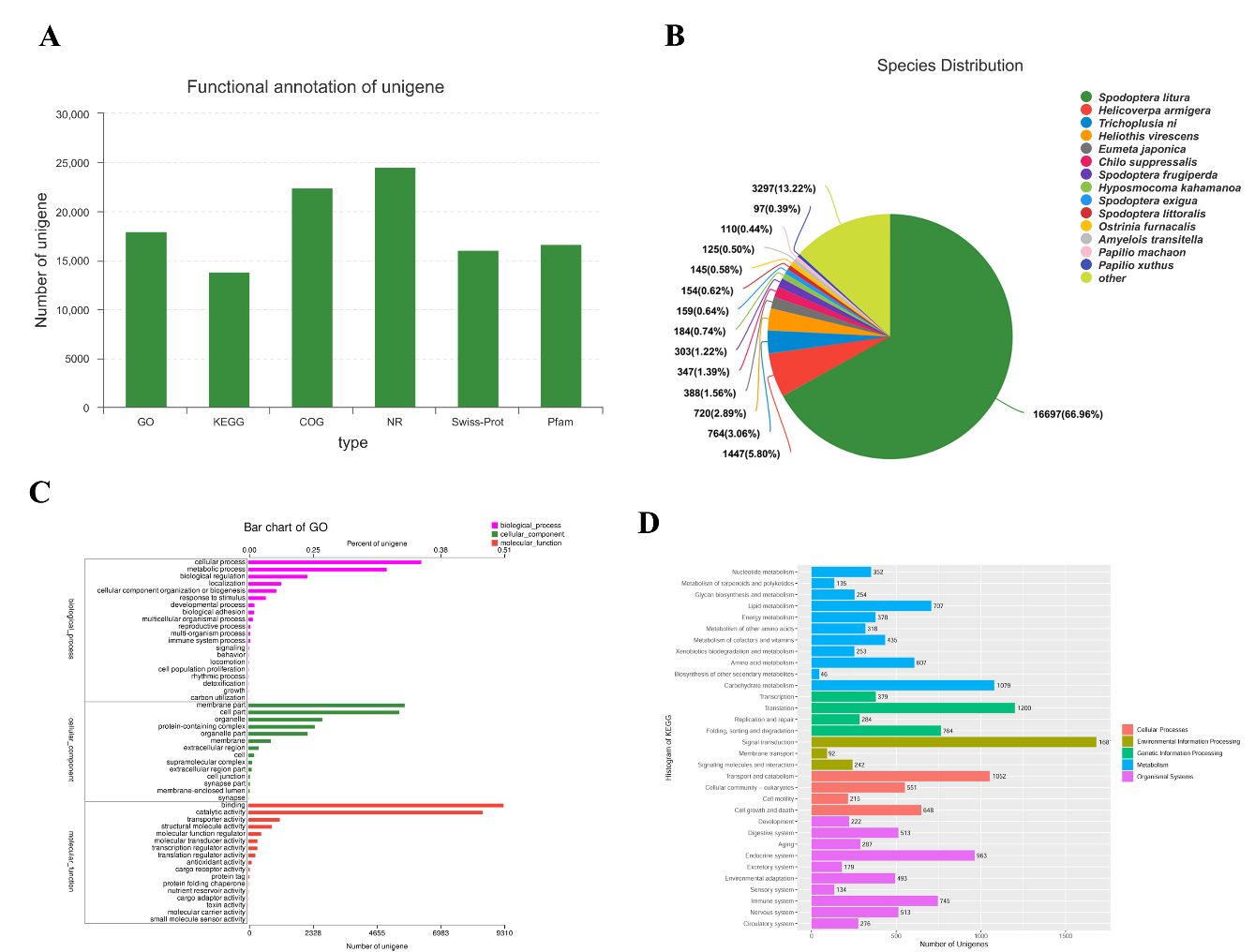


Supplement figure 1: Functional annotation of unigenes obtained from all the transcriptome data. A: The summary of unigene number annotated into NCBI NR, COG, GO, Pfam, Swiss-Prot and KEGG databases. B: The distribution of unigenes annotated to different Lepidoptera species. The top five species with most unigenes annotated to were *Spodoptera litura*, *Helicoverpa armigera*, *Trichoplusia ni*, *Heliothis virescens*, and *Eumeta japonica*. C: Gene Ontology (GO) of unigenes identified from the transcriptome data. D: Kyoto Encyclopedia of Genes and Genomes (KEGG) classification of unigenes obtained from the transcriptome data.


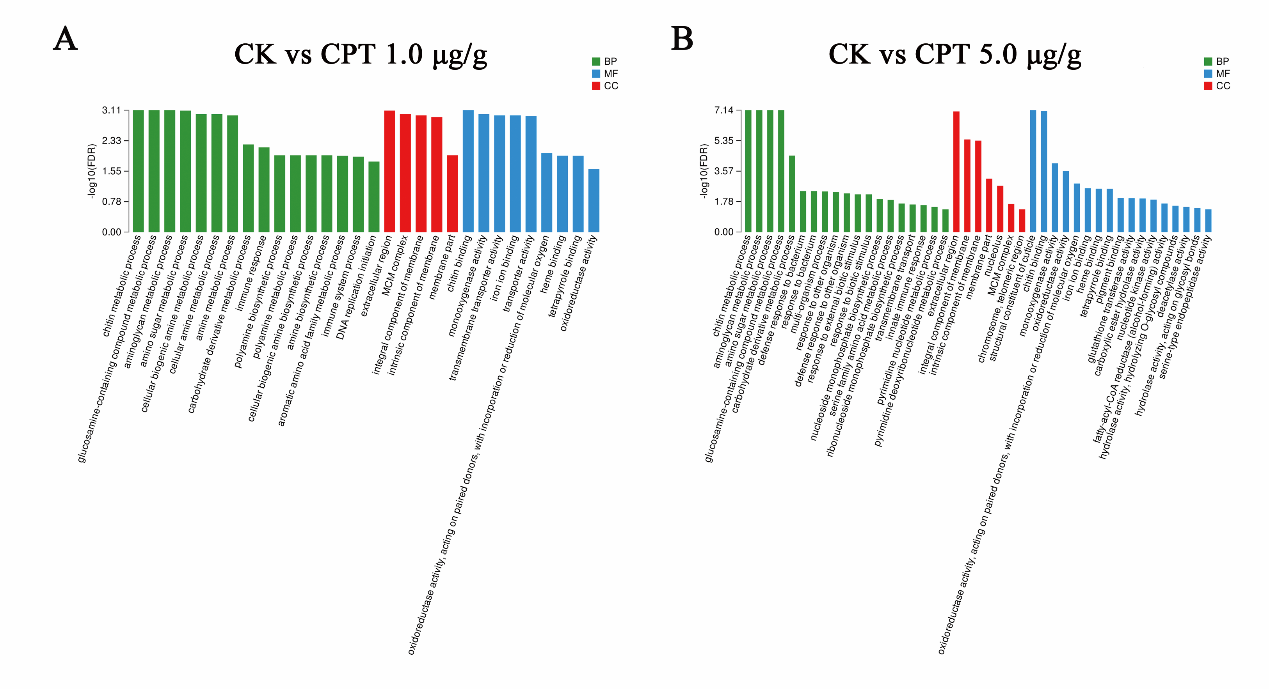
 Supplement Figure 2. Gene Ontology (GO) analyses of identified DEGs. A: The GO terms enriched significantly (corrected *P*-value < 0.05) in midgut samples from larvae treated with 1.0 μg/g CPT. B: The GO terms enriched significantly (corrected *P*-value < 0.05) in midgut samples from larvae treated with 5.0 μg/g CPT. GO terms were classified into three main categories: biological process, cell composition and molecular function.
